# Hepatitis C Virus Replication and Golgi Function in Brefeldin A-Resistant Hepatoma-Derived Cells

**DOI:** 10.1371/journal.pone.0074491

**Published:** 2013-09-18

**Authors:** Rayan Farhat, Lucie Goueslain, Czeslaw Wychowski, Sandrine Belouzard, Lucie Fénéant, Catherine L. Jackson, Jean Dubuisson, Yves Rouillé

**Affiliations:** 1 Inserm U1019, CNRS UMR8204, Center for Infection and Immunity of Lille (CIIL), Institut Pasteur de Lille, Université Lille Nord de France, Lille, France; 2 Institut Jacques Monod, CNRS UMR 7592, Université Paris Diderot, Sorbonne Paris Cité, Paris, France; University of North Carolina School of Medicine, United States of America

## Abstract

Recent reports indicate that the replication of hepatitis C virus (HCV) depends on the GBF1-Arf1-COP-I pathway. We generated Huh-7-derived cell lines resistant to brefeldin A (BFA), which is an inhibitor of this pathway. The resistant cell lines could be sorted into two phenotypes regarding BFA-induced toxicity, inhibition of albumin secretion, and inhibition of HCV infection. Two cell lines were more than 100 times more resistant to BFA than the parental Huh-7 cells in these 3 assays. This resistant phenotype was correlated with the presence of a point mutation in the Sec7 domain of GBF1, which is known to impair the binding of BFA. Surprisingly, the morphology of the cis-Golgi of these cells remained sensitive to BFA at concentrations of the drug that allowed albumin secretion, indicating a dichotomy between the phenotypes of secretion and Golgi morphology. Cells of the second group were about 10 times more resistant than parental Huh-7 cells to the BFA-induced toxicity. The EC_50_ for albumin secretion was only 1.5–1.8 fold higher in these cells than in Huh-7 cells. However their level of secretion in the presence of inhibitory doses of BFA was 5 to 15 times higher. Despite this partially effective secretory pathway in the presence of BFA, the HCV infection was almost as sensitive to BFA as in Huh-7 cells. This suggests that the function of GBF1 in HCV replication does not simply reflect its role of regulator of the secretory pathway of the host cell. Thus, our results confirm the involvement of GBF1 in HCV replication, and suggest that GBF1 might fulfill another function, in addition to the regulation of the secretory pathway, during HCV replication**.**

## Introduction

The replication of single-stranded positive RNA viruses occurs in association with rearranged intracellular membranes. For the hepatitis C virus (HCV) these membrane rearrangements have been named membranous web. Different types of HCV-induced membrane structures have been observed depending on the experimental model. The membranous web was initially described in U-2 OS cells inducibly expressing the HCV polyprotein [1], indicating that its formation depends on HCV protein expression, even without RNA replication. It was composed of small vesicles embedded in a membrane matrix. Similar membrane alterations were later observed in Huh-7 cells harboring a subgenomic replicon of genotype 1b [2] and in JFH1-infected Huh-7 cells [3]. In replicon-containing cells, it was reported to contain the nonstructural proteins NS3/4A, NS4B, NS5A and NS5B, and the genomic RNA [2]. Moreover, newly synthesized viral RNA was also detected in the membranous web, clearly indicating that it is a site of viral RNA synthesis [2].

In addition to the membranous web, a second type of HCV replicase was observed in Huh-7 cells containing a GFP-tagged replicon. This second type of replicase was made of smaller structures much more mobile than the membranous web, and scattered throughout the cell [4]. In highly permissive Huh-7.5 cells replicating a subgenomic replicon of the JFH1 strain at high levels, the membrane alterations were shown to be much more extensive, with the occurrence of numerous double membrane vesicles and of multivesicular structures [5] that had not been observed before with replicons of genotype 1b. These double membrane vesicles, together with single membrane vesicles were also observed in JFH1-infected Huh-7.5 or Lunet cells [6], [7]. It is unclear whether the difference of morphological alterations observed in these various studies primarily results from the host cell, the viral genotype or both.

The formation and the functioning of the membranous web are still poorly understood. Two viral proteins, NS4B and NS5A, appear to play a major role in the induction of membrane rearrangements [1], [6]. Based on morphological data showing a close association between the ER and the HCV replicases [1], [4]–[6], , and on biochemical data indicating that HCV RNA replication takes place in a compartment that sustains endoglycosidase H-sensitive glycosylation [9], the membranous web was proposed to be derived from the ER membrane. However, several endosomal markers were also observed colocalizing with HCV replicases and/or functionally involved in RNA replication [6], [10]–[12]. One major host factor implicated in HCV RNA replication is the phosphatidyl-inositol-4 kinase-IIIα (PI4KIIIα, also known as PI4KA) [11]–[16], an enzyme of the ER, which interacts with, and is activated by NS5A during HCV replication [16]–[18]. Its depletion by RNA interference leads to morphologically aberrant NS5A-positive structures in cells expressing the HCV polyprotein [6], [12], [18].

Recently, we and others found a role for the GBF1-Arf1-COP-I pathway in HCV replication [12], [19]–[21]. GBF1 is a guanine nucleotide exchange factor (GEF), which is recruited to the membrane of the cis-Golgi and activates the G-protein Arf1. Once activated by the binding of GTP, Arf1 in turn recruits different effectors, including the coat protein complex COP-I, which then forms vesicular carriers. This pathway mediates the retrograde transport from the cis-Golgi and the ERGIC to the ER [22], [23], and is also implicated in the biogenesis of lipid droplets [24]–[26]. Its inhibition by brefeldin A induces the collapse of the Golgi complex, the redistribution of Golgi glycosidases to the ER, and the block of the secretory pathway at the level of the ER exit. The precise role of this pathway in HCV replication is still unclear. Several other single-stranded positive RNA viruses of the *Picornaviridae* and *Coronaviridae* families also rely on this pathway for their replication [27]–[29]. The inhibition of HCV replication by brefeldin A is much stronger at the beginning of the infection than when the infection is already established [19], suggesting a crucial role at the onset of replication. The other GBF1-dependent RNA viruses, which do not establish chronic infections unlike HCV, do not share this special requirement for GBF1 early during infection. GBF1 does not appear to be a component of HCV replication complexes, because it did not co-localize with NS5A, a marker of HCV replication complexes, in confocal microscopy. The formation of membranous web-like structures in U-2 OS cells expressing the HCV polyprotein was not inhibited by brefeldin A, suggesting a role of the GBF1-Arf1-COP-I pathway in the functioning of the replication complexes rather than in their formation. Similarly, GBF1 does not seem to be required for the formation of membranous replication complexes of other single-stranded positive RNA viruses, such as poliovirus [27], and mouse hepatitis virus [28].

To gain more insight into the role of this pathway during HCV replication, we generated Huh-7-derived cell lines resistant to brefeldin A. We analyzed HCV replication and membrane traffic in these cells lines, and found that they can be classified into two phenotypes, one of which results from a mutation in the coding sequence of GBF1.

## Materials and Methods

### Chemicals

Dulbecco’s modified Eagle’s medium (DMEM), phosphate-buffered saline (PBS), trypsin-EDTA, OptiMEM, Oligofectamine, goat serum, and fetal calf serum (FCS) were purchased from Life Technologies. 4′,6-diamidino-2-phenylindole (DAPI) was from Molecular Probes. Golgicide A and Mowiol 3–88 were from Calbiochem. Protease inhibitors cocktail Complete was from Roche. Other chemicals were from Sigma.

### Antibodies

Mouse anti-E1 MAb A4 [30] was produced in vitro by using a MiniPerm apparatus (Heraeus) as recommended by the manufacturer. Sheep anti-NS5A antiserum [31] was kindly provided by Dr M. Harris (University of Leeds). Mouse anti-CD71 (transferrin receptor) MAb was purchased from Santa Cruz Biotechnology. Sheep anti-TGN46 was from Serotec. Mouse anti-GBF1, anti-EEA1, and anti-GM130 MAbs were from Transduction Laboratories. Rabbit anti-BIG1 was from Bethyl Laboratories. Mouse anti-Arf1 MAb 1D9 was from Abcam. Mouse anti-β tubulin MAb (TUB 2.1) was from Sigma. Mouse anti-HSA (ZMHSA1) was from Invitrogen. Goat anti-HSA (507313) was from Calbiochem. Goat anti-ApoE was from Millipore. Alexa 555-conjugated donkey anti-sheep IgG antibody was from Invitrogen. Peroxidase-conjugated goat anti-mouse, anti-rabbit, and anti-sheep IgG, and cyanine 3-conjugated goat anti-mouse IgG were from Jackson Immunoresearch.

### Cell Culture

Huh-7 [32], and MDCK (kindly provided by Dr Véronique Fafeur, Institut de Biologie de Lille, France) cells were grown in Dulbecco’s modified Eagle’s medium (DMEM), high glucose modification, supplemented with glutamax-I, sodium pyruvate, and 10% heat-inactivated fetal bovine serum. Huh-7-derived, BFA-resistant cells were selected and grown in the presence of BFA (40 to 100 ng/ml) in the same culture medium.

### HCVcc

The virus used in this study (JFH1-CSN6A4) was based on JFH1 [33], and contained cell culture adaptive mutations [34] and a reconstituted A4 epitope in E1, as described previously [19]. Luciferase-based assays were performed using an HCVcc containing the same cell culture adaptive mutations and expressing the *Renilla* luciferase reporter HCVcc-Rluc, as previously described [35]. An in-frame deletion introduced in the core-coding region of the HCVcc-Rluc construct was as previously described [36]. Nonreplicative control contained a GND mutation in the NS5B active site, as previously reported [33].

For the HCVcc infection assay, sub-confluent naïve Huh-7 cells grown in a 24-well plate were incubated with 200 µl of HCVcc-Rluc in the presence of BFA for 2 hours at 37°C, and the inoculate was replaced with fresh culture medium. Infected cells were cultured in the presence of BFA up to 6 hpi. Cells were lysed with 50 ul of 1× Renilla lysis buffer (Promega) at 24 hpi and the infection was scored by the measure of luciferase activity using a Renilla Luciferase Assay kit, as indicated by the manufacturer (Promega).

For the replication assay, cells were electroporated with HCVcc-Rluc/Δcore or HCVcRluc/GND RNA, and plated in 24-well plates in the presence of increasing concentrations of BFA. The BFA was removed 8 h later and the cells were further cultured without BFA. The luciferase activity was measured 4 h, 24 h, 48 h and 72 h post-electroporation. Luciferase activity at 4 hpi was expressed as 1 and the measures at other time points were normalized accordingly.

### RNA Interference

RNA interference experiments were carried out with pools of four different synthetic double-stranded siRNAs to the same target (on-target plus smart pool reagents from Dharmacon), as previously described [19]. A single non-targeting siRNA (D-001810-01), which has no impact on HCV infection, was used as a negative control. Briefly, sub-confluent cultures of Huh-7 cells in six-well clusters were transfected with 80 pmol of siRNA complexed with 4 µl of Oligofectamine in a total volume of 1 ml of OptiMEM per well for 6 h. Cells were trypsinized 24 h after siRNA transfection, plated in 24-well clusters, and analyzed by immunofluorescence 3 days after siRNA transfection.

For quantifying HCVcc infection, siRNA-treated cells were infected 24 h after trypsinization. Just before infection, extra wells of cells treated with each siRNA were counted to ensure that equal numbers of cells were infected. Relative expression levels of targeted proteins were analyzed by immunoblotting equal amounts of cell lysates. HCVcc infection was quantified by measuring NS5A expression levels by immunoblotting at 30 hpi.

### Immunofluorescence

Cells were processed for indirect immunofluorescent detection of viral proteins and cellular markers as previously described [3]. Images were acquired with an LSM710 confocal microscope (Zeiss) using a 63×/1.4 numerical aperture oil immersion objective. Signals were sequentially collected by using single fluorescence excitation and acquisition settings to avoid crossover. Images were assembled using Adobe Photoshop software.

### Immunoblotting

Cells were lysed in lysis buffer (50 mM Tris-Cl buffer pH 7.5 containing 100 mM NaCl, 1 mM EDTA, 1% Triton X-100, 0.1% sodium dodecyl sulfate, and protease inhibitors) for 20 min on ice. Cells were collected, and the nuclei were pelleted. The protein concentration in the postnuclear supernatants was determined by the bicinchoninic acid method as recommended by the manufacturer (Sigma), using bovine serum albumin as standard. Proteins were separated by SDS-polyacrylamide gel electrophoresis and transferred to nitrocellulose membranes (Hybond-ECL; Amersham) by using a Trans-Blot apparatus (Bio-Rad). The proteins of interest were revealed with specific primary antibodies, followed by species-specific secondary antibodies conjugated to peroxidase, and enhanced chemiluminescence detection as recommended by the manufacturer (Thermofischer). Signals were recorded with a LAS3000 imager (Fujifilm). Unsaturated signals were quantified with the gel macro of the ImageJ software.

### Viability Assay

Sub-confluent cell cultures grown in 96-well plates were incubated with BFA for 24 h. An MTS [3-(4,5-dimethylthiazol-2-yl)-5-(3-carboxymethoxyphenyl)-2-(4-sulfophenyl)-2H-tetrazolium]-based viability assay (CellTiter 96 aqueous nonradioactive cell proliferation assay from Promega) was conducted as recommended by the manufacturer.

### Albumin Secretion Assay

Sub-confluent cell cultures grown in 12-well plates were incubated for 24 h in 1 ml of complete culture medium containing BFA. Culture media were collected and centrifuged to remove cells debris. Cells were rinsed with PBS, and lysed in lysis buffer for 20 min on ice. The HSA concentration in the supernatants and lysates was determined by ELISA, using human serum albumin (HSA) as standard, as described [37]. The percentage of secretion was calculated as the percentage of HSA in the medium divided by the total amount of HSA in the medium and the lysate.

### GBF1 Sequencing

Total RNA was extracted using the NucleoSpin RNA II kit, as described by the manufacturer (Macherey-Nagel). GBF1 cDNA was obtained using the Expand Reverse Transcriptase and amplified by PCR using the High Fidelity DNA polymerase (Roche). Overlapping PCR products were purified in 1% agarose gel and were sent to Genoscreen to perform Sanger sequencing. The sequences of the primers used for the reverse transcription step (RT1 to 3) and the PCR and sequencing steps are described in table S1.

### Transfection

Huh-7 cells were transfected with expression vectors for yellow fluorescent protein (YFP), YFP-tagged inactive mutant (E794K), or BFA-resistant mutant (M832L) GBF1 [38] using the transfection reagent TransIT-LT1, as recommended by the manufacturer (Mirus).

## Results

### Isolation of Huh-7-derived Cell Lines Resistant to Brefeldin A

BFA-resistant cells were selected by cultivating Huh-7 cells in the presence of low doses of BFA. Most of the cells died in less than a week. Cells that survived were grown and passaged in the continuous presence of BFA for at least 2 months before characterization. Seven independent cell lines (named R1 to R7) were selected (table 1).

**Table 1:** Selection of BFA-resistant cell lines.

Cell line	Cell source	BFA (ng/ml)
R1	Huh-7	40
R2	Huh-7	100
R3	Huh-7	100
R4	Huh-7	50
R5	Huh-7	100
R6	Huh-7	100
R7	Huh-7	100

**Figure 1:**
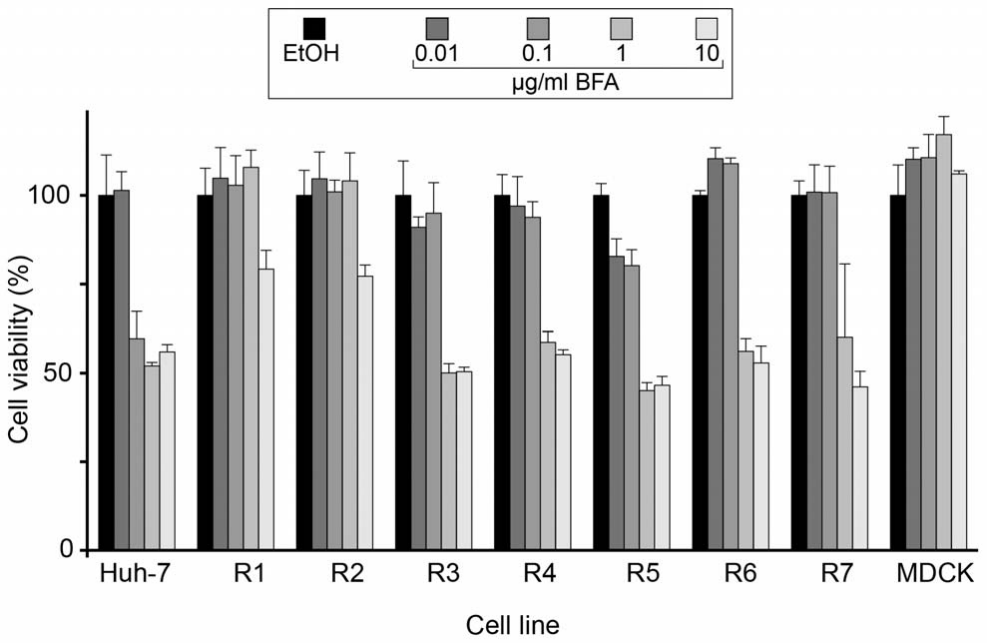
Viability of BFA-resistant cells. Sub-confluent cells of the indicated cell lines were cultured in 96-well plates in the presence of the indicated concentrations of BFA or of 0.2% ethanol (BFA stock solvent) for 24 h. Viability was assessed using an MTS assay. The absorbance of the ethanol-treated sample is expressed as 100%. Results were expressed as percentages of the values obtained with no BFA. Error bars represent the SEM of 2 independent experiments performed in triplicates.

The toxicity of BFA was assessed by measuring the cell viability after a 24 h-incubation in the presence of increasing concentrations of the drug. Cellular viability of Huh-7 cells decreased in the presence of 0.1 µg/ml of BFA or higher doses (figure 1). In two of the selected cell lines (R1 and R2), cells showed only a partial decrease (≈25%) of viability at the concentration of 10 µg/ml of BFA, with no impact of the lower doses (figure 1). For the other cell lines (R3–R7), the resistance to BFA was intermediate between those of parental Huh-7 cells and of resistant R1 and R2 cells. Their viability was reduced for the concentrations of 1 µg/ml and 10 µg/ml of BFA, with little or no effect of the concentration 0.1 µg/ml, even for the R4 cell line, which had been selected with a 50 ng/ml BFA concentration (figure 1). As expected, BFA had no effect on growth of MDCK cells [39], [40], which were used as a control in this experiment. These results allowed us to divide resistant cell lines into 2 groups depending on their degree of resistance to BFA. Cells of group 1 (R1 and R2) were more than 100 times more resistant to growth inhibition by BFA than Huh-7, and cells of group 2 (R3 to R7) were about 10 times more resistant.

### Action of BFA on HCV Infection in Resistant Cell Lines

**Figure 2:**
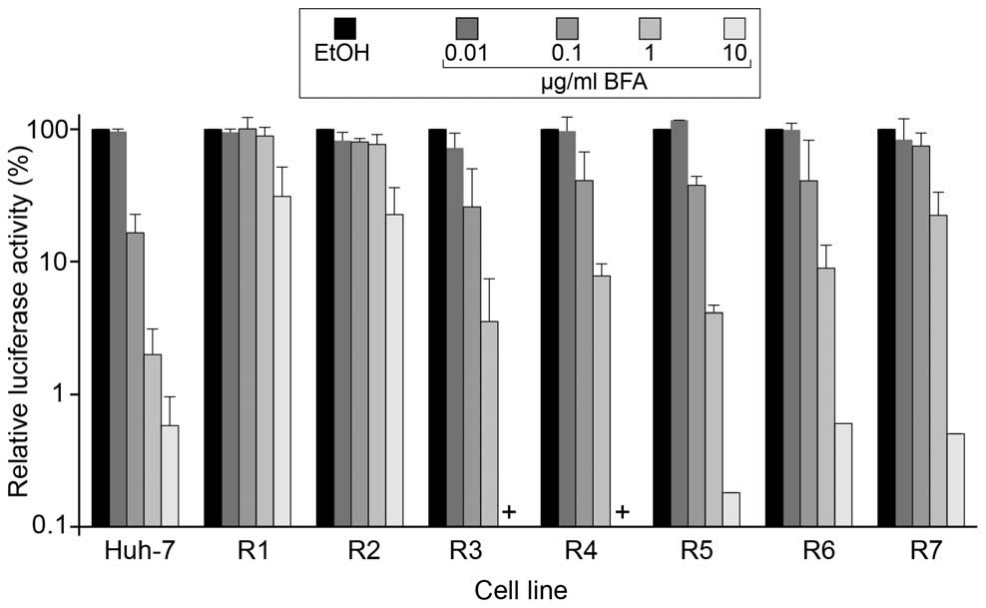
HCV infection in BFA-resistant cells. Cells of the indicated cell lines were infected for 2*Renilla* luciferase in the presence of the indicated concentrations of BFA. The virus was removed and the cells were left in the presence of BFA for another 6-h period. Cells were lysed in *Renilla* lysis buffer at 24 hpi, and the luciferase activity was quantified as a measure of HCV infection. Results were expressed as percentages of the values obtained with no BFA. Error bars represent the SD of 3 experiments performed in triplicates. +, values below 0.1%.

To assess the sensitivity of HCV infection to BFA in resistant cell lines, Huh-7 and resistant cell lines were infected with a recombinant HCVcc expressing the *Renilla* luciferase in the presence of increasing doses of BFA, and luciferase activity was quantified at 24 hpi. At this time post-infection, there are no re-infection events. This allows assessing the activity of the drug on the replication step of the HCV life cycle only. As previously reported [19], HCVcc infection in Huh-7 cells was inhibited in a dose-dependent manner, the lowest inhibitory concentration of BFA being 0.1 µg/ml (figure 2). Similar inhibitory patterns were observed in cells of group 2. Although the relative HCV infection was slightly higher in resistant cells of this group than in control Huh-7 cells with the doses of 0.1 and 1 µg/ml BFA, the EC_50_ were very similar for the R3 to R6 cell lines (in the range of 0.03–0.06 µg/ml) and for Huh-7 cells (≈0.04 µg/ml). Only R7 cells appeared slightly more resistant to HCV infection with an EC_50_ of ≈0.3 µg/ml. In contrast, HCVcc infection was much less susceptible to BFA in R1 and R2 cells, in which a partial inhibition of HCV infection was only observed with a concentration of 10 µg/ml (figure 2). Therefore, the resistant cells displayed dramatically different sensitivity to BFA with regard to HCVcc infection, group 1 cells being resistant to BFA inhibition of HCV infection, and group 2 cells sensitivity being very similar to that of Huh-7 cells.

BFA was previously shown to inhibit HCV infection at the replication step [19]. To confirm the resistance to BFA inhibition of HCV replication in R1 and R2 cells, we electroporated Huh-7, R1 and R2 cells with in vitro transcribed genomic RNA of an HCVcc construct expressing the *Renilla* luciferase and deleted from a major part of the core coding sequence in order to avoid BFA impact on viral egress and re-infection. Electroporated cells were treated for 8 hours with increasing concentrations of BFA, then the drug was removed and the luciferase activity was monitored as a measure of the replication. As previously described [19], the replication was inhibited by BFA in a dose-dependant manner in Huh-7 cells (figure S1). In contrast, the replication was insensitive to BFA in R1 and R2 cells, as shown by very similar curves of luciferase activity, regardless of the concentration of BFA (figure S1). These results indicate that the resistance to BFA inhibition of HCV infection in R1 and R2 cells results from a lack of action of BFA on the replication step of the HCV life cycle.

**Figure 3:**
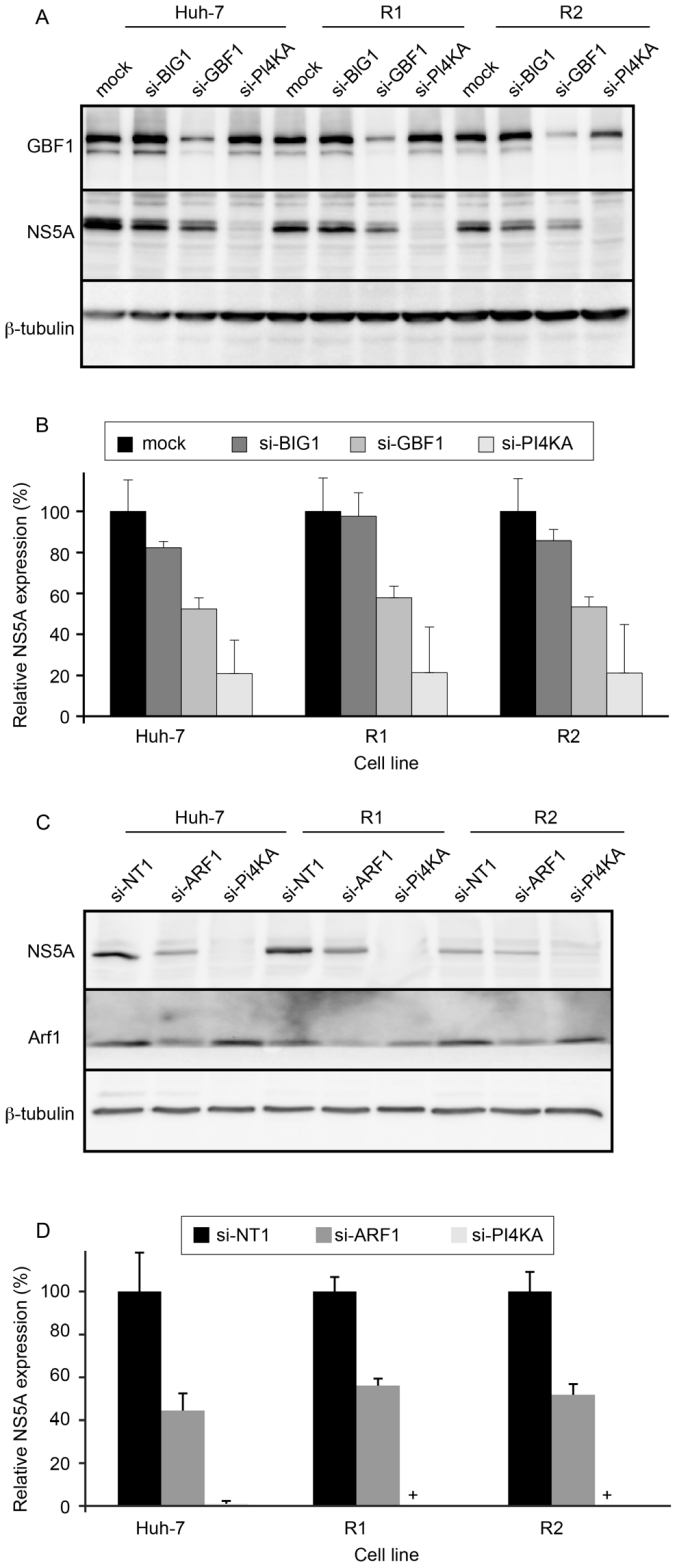
Impact of GBF1 and Arf1 depletion on HCV replication in R1 and R2 cells. (A) Cells of the indicated cell lines were transfected with siRNA targeting GBF1, BIG1, or PI4KIIIα (PI4KA), and infected with HCVcc. Cells were lysed at 30 hpi and the expression of NS5A, GBF1, and β-tubulin were analyzed by immunoblotting. (B) For each cell line, NS5A expression is expressed as a percentage of the values obtained for mock-transfected cells. (C) Cells of the indicated cell lines were transfected with siRNA targeting Arf1 or PI4KIIIα (PI4KA), or with a non-targeting control siRNA, and infected with HCVcc. Cells were lysed at 30 hpi and the expression of NS5A, Arf1, and β-tubulin were analyzed by immunoblotting. (D) For each cell line, NS5A expression is expressed as a percentage of the values obtained for the control siRNA. Error bars represent the SD of 3 independent experiments. +, NS5A below the detection limit.

We previously established that the BFA sensitivity of HCV infection results from the inhibition of GBF1 function [19]. In addition to GBF1, there are 14 other known Arf-GEFs in mammalian cells, most of them being BFA-insensitive [23]. Therefore, the resistance of HCV infection to BFA inhibition in R1 and R2 cells could have resulted from a shift of Arf-GEF usage from GBF1 to another BFA-insensitive Arf-GEF during HCV replication. To confirm that HCV infection in R1 and R2 cells was still dependent on GBF1, we performed siRNA-mediated depletion of GBF1 and analyzed the impact on HCV infection by quantifying NS5A expression. As previously reported [19], a reduction of about 70–80% of GBF1 expression induced an inhibition of HCV infection of about 50% in Huh-7 cells (figure 3A). The depletion of BIG1, another BFA-sensitive Arf-GEF not involved in HCV replication [19], only had a slight, non-significant effect, as compared to the untransfected control, whereas the depletion of the PI4KIIIα, a host factor essential for HCV replication, inhibited NS5A expression by about 80% in the same experimental setting (figure 3A). The levels of inhibition imposed by GBF1, BIG1 or PI4KIIIα depletions were very similar in Huh-7, R1 and R2 cells (figure 3B). These results indicate that, despite its resistance to BFA inhibition, HCV infection in R1 and R2 cells still depends on GBF1.

In addition to GBF1, HCV replication also requires Arf1 function [20], [21]. We therefore tested whether HCV replication in R1 and R2 cells was still dependent on Arf1, using siRNA-mediated depletion. Arf1-depletion resulted in about 50–60% inhibition of HCV infection in Huh-7 cells, as compared to cells transfected with a control non-targeting siRNA, despite an apparently moderate efficiency of Arf1 siRNA (figure 3C). However, the actual efficiency of Arf1 depletion is probably underestimated, since the anti-Arf1 antibody we used cross-reacts with other Arf family members. Very similar inhibitory patterns were observed in Huh-7, R1, and R2 cells (figure 3D), indicating that HCV infection depends on Arf1 in R1 and R2 cells.

Our results indicate that HCV infection of R1 and R2 resistant cells requires GBF1 and Arf1, as in parental, BFA-sensitive Huh-7 cells. This suggests that the absence of BFA inhibition could result from the selection of cells expressing a BFA-resistant form of GBF1 or Arf1.

### Action of BFA on Secretion in Resistant Cell Lines

**Figure 4:**
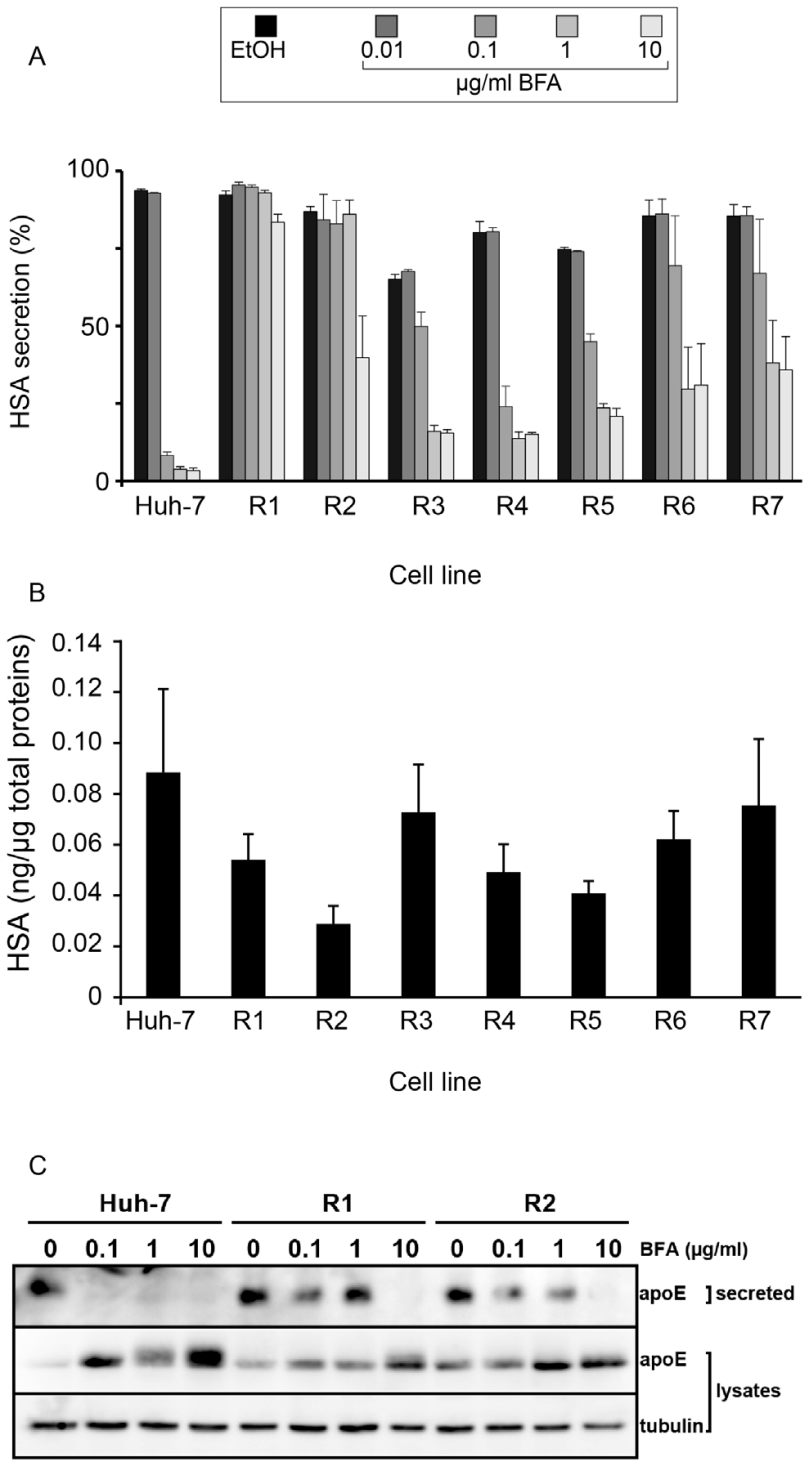
Serum albumin and apolipoprotein E secretion in BFA-resistant cells. (A) Albumin secretion. Cells of the indicated cell lines were seeded in 12-well plates, and cultured in the presence of BFA for 24 h. The amounts of human serum albumin (HSA) in the conditioned culture media and in cell lysates were quantified with an ELISA assay and expressed as percentages of HSA secretion. Error bars represent the SEM of 4 independent experiments performed in duplicates. (B) Basal HSA expression levels. HSA of the indicated cell lines were quantified from cell lysates (in the absence of BFA treatment) by ELISA and normalized to the total protein concentration. (C) Apolipoprotein E (apoE) secretion. Cells were cultured in 24-well plates, in the presence of the indicated concentrations of BFA for 8 h. The amounts of apoE in cell lysates and culture media and of tubulin in cell lysates were analyzed by immunoblotting. Error bars represent the SEM of 3 independent experiments.

Since BFA blocks secretion in a GBF1-dependent manner, we tested the functionality of the secretory pathway of the resistant cells in the presence of BFA. We quantified the secretion of human serum albumin (HSA) using an ELISA assay. HSA is strongly expressed in Huh-7 cells and is constitutively secreted. BFA blocks protein transport and has no effect on protein synthesis [41]; we therefore measured HSA in cell lysates and supernatants and quantified HSA secretion as the percentage of secreted protein during a 24-h period. HSA secretion was almost completely (>95%) blocked by BFA in Huh-7 cells at 0.1 µg/ml and higher concentrations (figure 4A).

The resistant cell lines expressed similar or reduced levels of albumin, as compared to Huh-7 cells (figure 4B). Concerning HSA secretion, the two groups of resistant cells again displayed different sensitivities to BFA. HSA secretion from R1 and R2 cells showed a small reduction at the concentration of 10 µg/ml BFA only, with no effect of the lower doses. In contrast, the cells of group 2 showed a partial residual HSA secretion at the concentration of 0.1 µg/ml and reached a maximal inhibition at 1 µg/ml (figure 4). The EC_50_ was shifted from 0.06 µg/ml in Huh-7 cells to 0.09–0.11 µg/ml in cells of the R3–R7 lines. However, the main difference with Huh-7 cells was the higher rate of secretion in the presence of 1 and 10 µg/ml of BFA. The residual albumin secretion at these concentrations of BFA was comprised between 18% (for R4) and 44% (for R7) of the secretion in the absence of BFA, whereas in Huh-7 cells the residual secretion was about 3%.

The reduced BFA-induced inhibition of protein secretion in R1 and R2 cells was confirmed with apolipoprotein E (apoE), which is a component of VLDL particles that are secreted by Huh-7 cells. Huh-7, R1 and R2 cells were treated for 8 hours with increasing concentrations of BFA, and the levels of apoE in cells and conditioned media were investigated by immunoblotting. As shown in figure 4C, apoE was absent from the medium of cells treated by BFA at 0.1 µg/ml or higher concentrations, and the levels of apoE were increased in the corresponding cell lysates, indicating an inhibition of the secretion of apoE. In contrast, apoE remained secreted from R1 and R2 cells in the presence of up to 1 µg/ml of BFA, and BFA only blocked apoE secretion at 10 µg/ml (figure 4C). These results confirmed the reduction of BFA-induced inhibition of secretion in R1 and R2 cells. Altogether, the data indicate the existence of different mechanisms of resistance to BFA in the two groups of cells, and suggest the presence of BFA-resistant forms of GBF1 in R1 and R2 cells.

### Identification of a Mutation in the Sec7 Domain of GBF1 in R1 and R2 Cells

The lack of BFA inhibition of secretion in R1 and R2 cells could result from a mutation that reduces the interaction of BFA with its target, the GBF1-Arf•GDP complex. To investigate this hypothesis, we sequenced the coding sequence of GBF1 and of the four known BFA-sensitive human Arfs (Arf1, Arf3, Arf4 and Arf5). Three isoforms of GBF1 have been reported [42]. We only detected the expression of GBF1 isoform 3 (Genbank accession number NM_001199379.1) in Huh-7 and Huh-7-derived cells. The complete GBF1 coding sequence was determined. Four point mutations were found, as compared to the reference sequence. These mutations led to amino-acid substitutions at positions 547, 1396, 1725, and 1833 of the coding sequence of GBF1 isoform 3 (table 2). All these mutations were present together with the wild type sequence (data not shown), suggesting that only one copy of the gene was mutated. One of these mutations changes the residue of tyrosine 547 for an asparagine in the HUS domain, the function of which is unknown [43]. The mutated Y547N residue is located two residues away from the highly conserved “HUS box”, which is the most highly conserved region of GBF/BIG GEFs outside of the Sec7 domain [43]. The three other mutations lie in non-conserved interdomain regions. The same set of mutations was found in Huh-7 cells and in cells from group 2 cells (data not shown). We therefore conclude that none of these mutations could account for the moderate resistance to BFA of these cells.

**Table 2:** Mutations found in the coding sequence of GBF1 in Huh-7 cells.

Reference sequence^a^		Amino acid sequence	
Position	Mutation	Position	Residue
1933	T/A	547	Tyr/Asn
4480	C/A	1396	Leu/Met
5469	G/T	1725	Glu/Asp
5791	G/A	1833	Ala/Thr

a Genbank NM_001199379.1.

**Figure 5:**
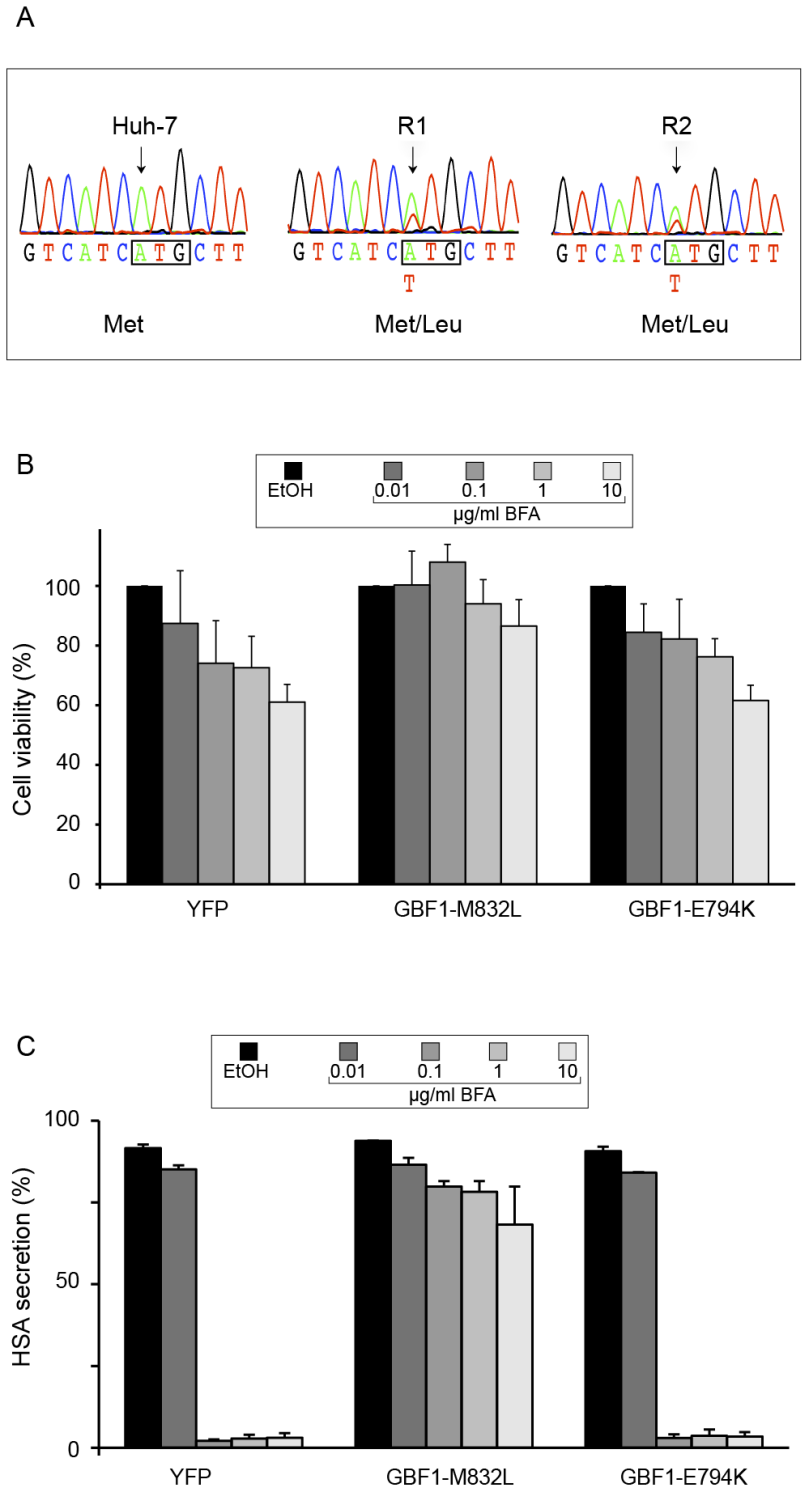
Mutation detected in GBF1 of R1 and R2 cells. (A) A fraction of the electrophoregrams corresponding to the sequence of GBF1 from the indicated cell lines is presented. The nucleotide and amino-acid sequences are indicated. The position of the mutation is indicated by an arrow. (B) Huh-7 cells were transfected with expression plasmids for GBF1-M832L, GBF1 inactive mutant E794K, or YFP. Transfected cells were submitted to a cell viability assay, as explained in the legend of figure 1. Results were expressed as percentages of the values obtained with no BFA. Error bars represent the SEM of 3 independent experiments performed in triplicates. (C) Transfected cells were seeded in 12-well plates, and cultured in the presence of BFA for 24 h. The amounts of human serum albumin (HSA) in the conditioned culture media and in cell lysates were quantified with an ELISA assay and expressed as percentages of HSA secretion. Error bars represent the SEM of 3 independent experiments performed in duplicates.

In contrast, we observed in R1 and R2 cells a mutation that was absent from GBF1 of Huh-7 and cells of group 2. It is a substitution of a thymine by an adenine at position 2788 of the sequence of reference. This mutation leads to the change of the methionine 832 ATG codon into a leucine TTG codon (figure 5A). This mutation affects a residue of the Sec7 domain, which is the domain interacting with Arf and with BFA. In both cell lines, the mutation was observed together with the wild type sequence, suggesting that only one copy of the GBF1 gene is mutated in R1 and R2 cells (figure 5A).

We also sequenced the Sec7 domains of the two other BFA-sensitive Arf-GEFs (BIG1 and BIG2) and the coding sequences of the 4 human Arfs known to be sensitive to BFA inhibition. No additional mutation was observed in any of the resistant cell lines (data not shown).

To test the functional impact of the M832L mutation, a YFP-tagged GBF1 construct containing this mutation was expressed in Huh-7 cells, and the cells were submitted to a BFA challenge. The GBF1-M832L construct induced a protection of the cells to the BFA-induced toxicity, as compared to control cells expressing YFP (figure 5B). In contrast, cells expressing an YFP-tagged GBF1-E794K inactive mutant did not respond differently than YFP-expressing control cells. Similarly, the expression of GBF1-M832L, but not GBF1-E794K, rescued HSA secretion in the presence of BFA (figure 5C). These data confirm the impact of the M832L mutation on BFA sensitivity of GBF1, and indicate that the M832L mutation present in the GBF1 of R1 and R2 cells is responsible for the BFA resistant phenotype of these cells.

### Impact of BFA on the Morphology of the Golgi and Endosomes

**Figure 6:**
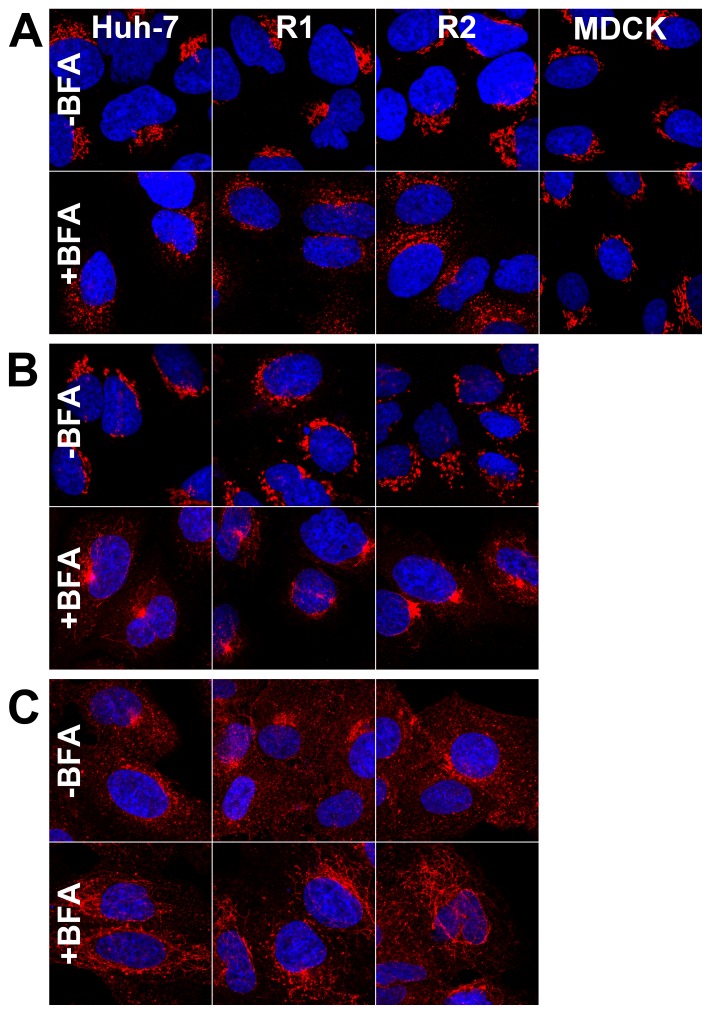
Morphology of BFA-sensitive compartments of R1 and R2 cells. Cells of the indicated cell lines were treated with 2.5 µg/ml (A) or 5 µg/ml (B, C) BFA for 30 minutes, fixed and processed for the immunofluorescent detection of GM130 (A), TGN46 (B), or the transferrin receptor (C). The nuclei were stained with DAPI. Representative confocal images are presented. Bar, 20 µm.

BFA treatment is known to alter the morphology of the Golgi and endosomes. To further analyze the impact of the M832L mutation, we investigated the sensitivity of these organelles to BFA in R1 and R2 cells. Huh-7 cells were incubated for 30 min in the presence of 5 µg/ml BFA and processed for immunofluorescent detection of the cis-Golgi marker GM130, the trans-Golgi network marker TGN46, and the transferrin receptor, a marker of recycling endosomes. As already reported for other cell lines, the cis-Golgi labeling was scattered in the presence of BFA (figure 6A), whereas the TGN structure was condensed into a perinuclear dot-like structure (figure 6B), and the recycling endosomes merged into a large network of tubules (figure 6C), also partially labeled with the TGN marker. As expected, EEA1, another endosomal marker, was unaffected by the BFA treatment (data not shown).

The morphological changes of the TGN and recycling endosomes were very similar in R1 and R2 cells (figures 6B and 6C). This sensitivity was expected, since it does not depend on GBF1, but rather on BIG1 and BIG2. Surprisingly, the pattern of GM130 labeling was scattered in R1 and R2 cells, as in Huh-7 cells. When the cells were treated for 30 min with a reduced concentration of 2.5 µg/ml BFA, the pattern of GM130 labeling was again similar in resistant cells and Huh-7 cells, whereas MDCK cells were clearly unaffected (figure 6A), indicating a sensitivity of the cis-Golgi morphology in these R1 and R2 cells, despite the M832L mutation of GBF1. A similar sensitivity of the cis-Golgi morphology to Golgicide A (25 µM), a specific inhibitor of GBF1 function, was also observed with R1 and R2 cells (data not shown).

**Figure 7:**
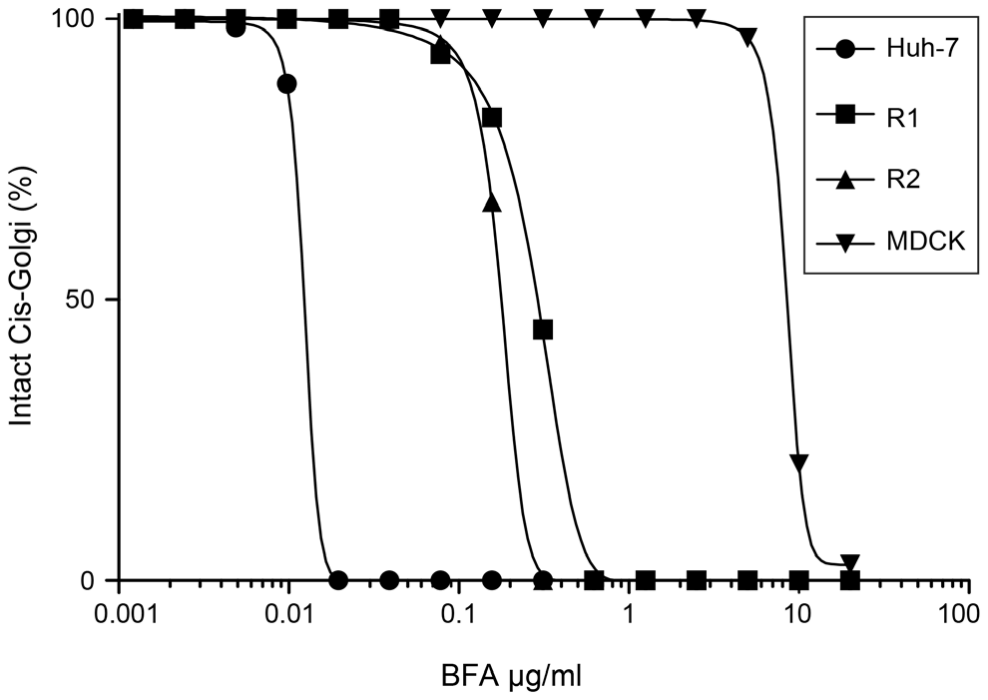
BFA sensitivity of the cis-Golgi of Huh-7, R1, R2 and MDCK cells. Cells were treated for 30 minutes with increasing concentrations of BFA, fixed and processed for the immunofluorescent detection of GM130. For each condition, approximately 100 cells were scored for their cis-Golgi morphology, as either intact or scattered. For each cell line, the percentages of cells with intact cis-Golgi morphology were plotted against BFA concentrations.

This BFA sensitivity of the morphology of the Golgi complex of R1 and R2 cells at BFA concentrations that did not inhibit HSA secretion was unexpected. To further characterize this process, we used a wide range of BFA concentrations to assess the relative sensitivities of the cis-Golgi of Huh-7, R1, R2 and MDCK cells. As shown in figure 7, all cells turned out to be responsive to BFA, however with dramatically different sensitivities. The EC_50_ was 12 ng/ml for Huh-7 cells, in the range of 200–300 ng/ml for R1 and R2 cells, and 8.5 µg/ml for MDCK cells (figure S2 and table 3). Interestingly, the morphology of the cis-Golgi appeared approximately 40–50 times more sensitive to BFA than the secretion of HSA in R1 and R2 cells, and only about 5 times in Huh-7 cells.

**Table 3:** Half maximal effective concentrations of BFA effects on the cis-Golgi morphology and HSA secretion.

Cell line	Golgi morphologyEC_50_ (ng/ml)	HSA secretionEC_50_ (ng/ml)
Huh-7	12	60
R1	280	>10000
R2	180	≈10000
MDCK	8500	ND

**Figure 8:**
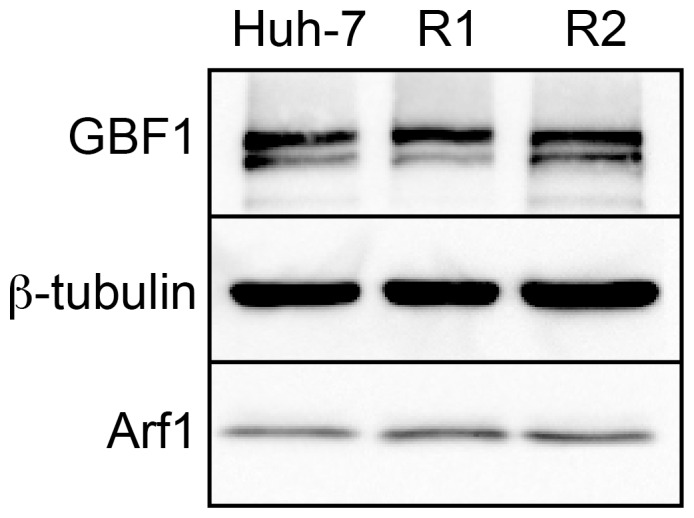
Immunoblot analysis of GBF1 and Arf1 expression in R1 and R2 cells. Equal amounts of lysates of the indicated cell lines were analyzed by immunoblotting for the expression of GBF1, Arf1 and β-tubulin.

We also compared relative GBF1 and Arf1 expression levels in Huh-7, R1 and R2 cells by immunoblotting. As shown in figure 8, Huh-7, R1 and R2 cells expressed similar levels of GBF1 and Arf1, indicating that the sensitivity of the Golgi morphology of R1 and R2 cells to BFA, despite the M832L mutation, does not result from increased or reduced expression levels of GBF1 or Arf1. Unfortunately we could not compare the expression levels in MDCK cells, because the antibody did not recognize GBF1 of MDCK cells. We also investigated the intracellular localization of GBF1 by immunofluorescence, and found no difference in the localization between R1, R2 and Huh-7 cells in the absence of BFA or in the presence of 5 µg/ml BFA (data not shown).

**Figure 9:**
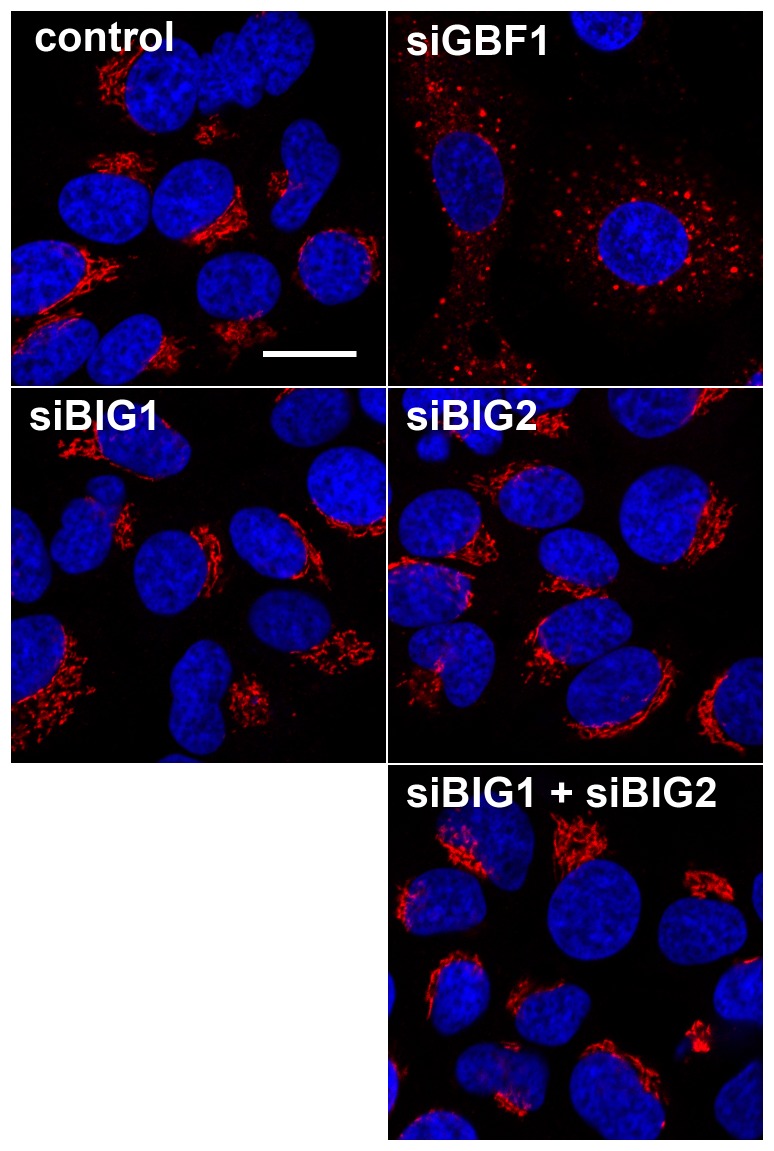
Impact of Arf-GEF depletion on the cis-Golgi morphology. Huh-7 cells were transfected with siRNA targeting GBF1, BIG1, BIG2, or both BIG1 and BIG2. Cells were fixed 3 days after transfection and processed for the immunofluorescent detection of GM130 (shown in red). The nuclei were stained with DAPI (shown in blue). Representative confocal images are presented. Bar, 20 µm.

To determine if the inhibition of other BFA-sensitive Arf-GEFs could participate in the change of the cis-Golgi morphology in response to BFA treatment, we used siRNA to specifically deplete GBF1, BIG1 and BIG2. As expected, the depletion of GBF1 induced a dramatic scattering of the GM130 labeling, very reminiscent of the effect of a BFA treatment (figure 9). In contrast, the depletion of BIG1, BIG2 or both BIG1 and BIG2 had no apparent effect on the pattern of GM130 labeling (figure 9), indicating that these two BFA-sensitive Arf-GEFs do not regulate the cis-Golgi morphology. Taken together, these results indicate a dichotomy between functional and morphological effects of the M832L mutation on the Golgi complex.

## Discussion

In this study, we selected Huh-7-derived, BFA-resistant cell lines and analyzed their phenotype regarding HCV infection and membrane traffic. Seven independent cell lines were generated using essentially the same selection protocol (except for the cell lines R1 and R4, which were selected using reduced concentrations of BFA), and two different phenotypes were obtained. Two cell lines (R1 and R2) were highly resistant to BFA. More than 100 times more BFA than in Huh-7 cells was required to observe similar effects regarding cell viability, albumin secretion, and HCV infection. On the other hand, the five other cell lines were only 10 times more resistant to BFA-induced toxicity, and about as sensitive as parental Huh-7 cells regarding HCV infection.

The resistance phenotype of the first group of cells (R1 and R2) was correlated with the presence of a point mutation in the Sec7 domain of GBF1, which replaces the methionine residue 832 with a leucine. The M832 residue is part of the BFA binding site at the Sec7-Arf interface. Crystal structures of the complex between the Sec7 domain, BFA and Arf-GDP have been reported for Sec7 domains of a modified, BFA-sensitive Arno [44], and of the yeast GBF1 homolog Gea1 [45]. In both crystal structures, the conserved methionine residue corresponding to M832 of GBF1 engages van der Waals contacts with BFA. Its substitution to a leucine residue has been reported to render GBF1 resistant to BFA in a dominant manner [38], [46]. Accordingly, expressing a GBF1-M832L construct in Huh-7 cells resulted in a phenotype of increased resistance to both BFA-induced toxicity and inhibition of HSA secretion. Moreover, we previously showed that the expression of GBF1-M832L renders Huh-7 cells resistant to the BFA-induced inhibition of HCV infection [19]. Therefore, the presence of this mutation in R1 and R2 cells is sufficient to explain the BFA-resistant phenotype of R1 and R2 cells.

The phenotype of the second group of cells (R3– R7) was not associated with any mutation in the coding sequence of GBF1, of BFA-sensitive Arfs, or of the Sec7 domains of BIG1 and BIG2. The resistance to BFA-induced toxicity was only slightly increased, and could result from increased compensatory mechanisms in selected cells such as a reduced induction of the unfolded protein response (UPR) and/or of UPR-induced apoptosis in response to BFA [47]. The unusual pattern of inhibition of albumin secretion in the presence of increasing doses of BFA again suggests the existence of compensatory mechanisms, allowing the secretory pathway of these cells to be partially functional at concentrations of BFA that are fully inhibitory for Huh-7 cells. The nature of these putative compensatory mechanisms must await further studies.

Surprisingly, although the secretory pathway of R1 and R2 cells was functional, we consistently observed at BFA concentrations comprised between 1 and 10 µg/ml a disruption of their cis-Golgi that appeared very similar to what was observed in BFA-sensitive Huh-7 cells. This indicates that the secretory pathway of cells with a BFA-resistant GBF1 is functional in the presence of BFA, in spite of a dramatically disorganized Golgi complex morphology, including the scattering of the cis-Golgi and the collapse of the trans-Golgi network. Our results clearly indicate a dichotomy between BFA impacts on the cis-Golgi scattering and the inhibition of HSA secretion. Even in parental, BFA-sensitive Huh-7 cells, the two effects of BFA were observed at different concentrations. Interestingly, no clear correlation between Golgi morphology and functional secretion was also found in a recent siRNA screen of regulators of Golgi architecture [48]. As previously reported [49], the scattering of the cis-Golgi marker was also observed after siRNA-mediated depletion of GBF1. This is consistent with a role of GBF1 in the regulation of the Golgi morphology, as reported previously [50], [51]. Nonetheless, the sensitivity of the Golgi morphology to BFA in R1 and R2 cells with a BFA-resistant GBF1 due to the M832L mutation was more puzzling, especially since MDCK cells, which also have a leucine residue instead of a methionine at position 832 of GBF1, did not appear to have a Golgi morphology sensitive to BFA in the same experimental conditions. However, we found that the morphology of the cis-Golgi of MDCK cells is also sensitive to BFA to some extent. This suggests that the M832L mutation reduces but does not completely abolish the binding of BFA to GBF1. This is consistent with the observation that BFA engages contacts with other residues of the binding site of GBF1 [38], [46].

The difference of sensitivity of R1/R2 cells and of MDCK cells remains unexplained. The fact that MDCK cells express GBF1-M832L only, when R1 and R2 cells probably express both the wild type and the mutant protein, could play a role in this difference of sensitivity. We cannot exclude the possibility that differences between the sequences of the proteins of dog and human origin could also contribute to this difference of sensitivity. Additional BFA-sensitive factor might also regulate the morphology of the cis-Golgi with no impact on secretion. A role for BIG1 as an additional BFA-sensitive factor in the regulation of the cis-Golgi morphology of HeLa cells has recently been proposed [52]. However, we did not observe any impact of BIG1 depletion on the cis-Golgi morphology of Huh-7 cells.

The precise function of GBF1 during HCV replication is still elusive. GBF1 has been reported to be involved in the replication of several positive RNA viruses of the *Picornaviridae, Coronaviridae* and *Flaviviridae* families [19], [27]–[29]. For all these viruses, the replication of the genomic RNA takes place in vesicular, rearranged membranes originating, at least in part, from membranes of the early secretory pathway. However, for all these families of viruses, GBF1, which is a major regulator of the early secretory pathway, appears to have no major role in the formation of the rearranged membranes of infected cells, but rather be involved in the functioning of these structures [19], [27], [28]. We previously hypothesized that GBF1-associated mechanisms might function to deliver proteins or lipids to HCV replication complexes [19]. Our results with the cells of group 2 suggest that the role of GBF1 in HCV replication does not simply reflect its role as a regulator of the protein secretory pathway. Indeed, the pattern of BFA inhibition of HSA secretion does not match the one of HCV infection in these cell lines. HCV infection is completely inhibited by 1 µg/ml BFA, whereas HSA secretion occurs at a rate comprised between 18 and 44% of its secretion in the absence of BFA. This suggests that compensatory mechanisms exist in these cells that allow HSA to be transported out of the ER in the presence of BFA, since this is the main step of the secretory pathway that is inhibited by BFA. However these compensatory mechanisms do not support the replication of HCV, even at a reduced rate. This suggests that GBF1 function during HCV replication does not only reflect the functioning of the secretory pathway, but it could also fulfill an additional function during HCV replication. This additional function could be either related to another cellular function of GBF1, or GBF1 could be, at least in part, diverted from its normal cellular functions during HCV infection. It is worth noting that the inhibition of poliovirus replication by BFA can be largely rescued by expression of only the N-terminus of GBF1, not including the catalytic Sec7 domain [53]. This indicates the existence of a GBF1 function unrelated to Arf1 activation during poliovirus replication. It would be interesting to determine whether a similar situation could be observed with HCV. We and others have shown that Arf1 is required for HCV replication. However, this does not preclude the existence of an additional GBF1 function unrelated to Arf1 activation during HCV replication. It would be interesting to determine whether GBF1 mutants that do not localize to the Golgi could rescue an infection inhibited by BFA or by GBF1 depletion. In addition to GBF1, BIG1 and BIG2 have been suggested to be involved in the replication of poliovirus [54]. The absence of inhibition of HCV infection by BFA in R1 and R2 cells strongly suggests that GBF1 is the only BFA-sensitive factor required for HCV infection. This indicates that BIG1 and BIG2 are not required for HCV entry or replication.

It has been suggested that GBF1 and Arf1 would be involved in the formation of PI4P in the replication complexes through the trafficking of the PI4 kinase PI4KIIIβ [21]. The formation of PI4P by PI4KIIIβ in infected cells has been shown to be essential for the replication of several positive RNA viruses [55]. For HCV, several groups have provided compelling evidence for the involvement of PI4KIIIα in HCV replication [11]–[16]. In contrast, the function of PI4KIIIβ in HCV replication is still controversial [15], [16], [18]. It has been shown that NS5A is a critical inducer of PI4P formation through its interaction and activation of PI4KIIIα [16], [18]. Whether GBF1 and Arf1 contribute to this process is still unknown. It was recently reported that the PI4P produced by PI4KIIIαmodulates the recruitment of GBF1 by rab1b to the Golgi membrane [56].

In addition to GBF1, Arf1, and COP-I, other regulators of membrane dynamics in the early secretory pathway, including rab1b [57] and its negative regulator TBC1D20 [58], [59], have been reported to be involved in the replication of HCV. It is not yet known if these host factors regulate the formation or the functioning of the membranous web, and if their function is related to the one of GBF1 during HCV replication. However it is striking that rab1b is a GBF1-binding partner, which modulates its function in the secretory pathway of non-infected cells [60]. Therefore, even though the function of GBF1 in HCV replication appears to be different from its role of regulator of the secretory pathway, it is tempting to speculate that the virus subverts the whole rab1-GBF1-Arf1-COP-I pathway, normally devoted to the regulation of membrane dynamics in the secretory pathway, for the functioning of the membranous web.

## Supporting Information

Figure S1 **BFA does not inhibit HCV replication in R1 and R2 cells.** Huh-7 cells (A), R1 cells (B), and R2 cells (C) were electroporated with HCVcc-Rluc/Δcore or HCVcc-Rluc/GND RNA. HCVcc-Rluc/Δcore-electroporated cells were incubated for 8 h in the presence of the indicated concentrations of BFA. Cells were lysed in *Renilla* lysis buffer at indicated time points post-electroporation, and the luciferase activity was quantified as a measure of HCV replication. Results were normalized to the values obtained at 4 hpi with no BFA.
(TIF)

Figure S2 **Impact of BFA on the cis-Golgi morphology of MDCK cells.** MDCK cells were treated for 30 minutes with the indicated concentrations of BFA, fixed and processed for the immunofluorescent detection of GM130 (shown in red). The nuclei were stained with DAPI (shown in blue). Representative confocal images are presented. Bar, 20 µm.
(TIF)

Table S1 **Primers used for GBF1 sequencing.**
(DOCX)
