# Supplementary figures and images for: Hepatitis C Virus Replication and Golgi Function in Brefeldin A-Resistant Hepatoma-Derived Cells
Source: PLoS One. 2013 Sep 18;8(9):e74491. doi: 10.1371/journal.pone.0074491 (PMC3776844; doi:10.1371/journal.pone.0074491)

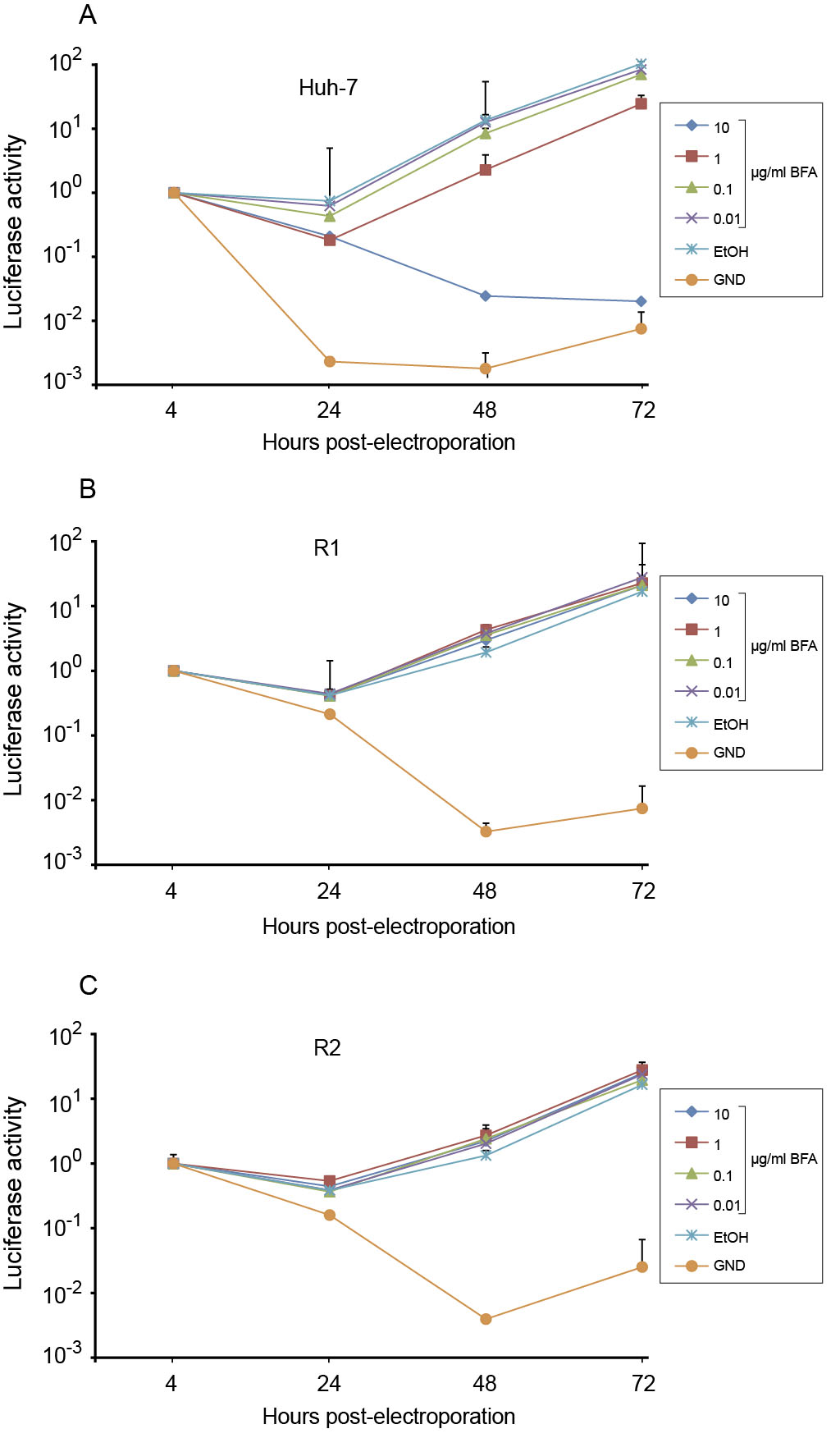

Supplement: Figure S1 — BFA does not inhibit HCV replication in R1 and R2 cells. Huh-7 cells (A), R1 cells (B), and R2 cells (C) were electroporated with HCVcc-Rluc/Δcore or HCVcc-Rluc/GND RNA. HCVcc-Rluc/Δcore-electroporated cells were incubated for 8 h in the presence of the indicated concentrations of BFA. Cells were lysed in Renilla lysis buffer at indicated time points post-electroporation, and the luciferase activity was quantified as a measure of HCV replication. Results were normalized to the values obtained at 4 hpi with no BFA. (TIF) [file pone.0074491.s001.tif]

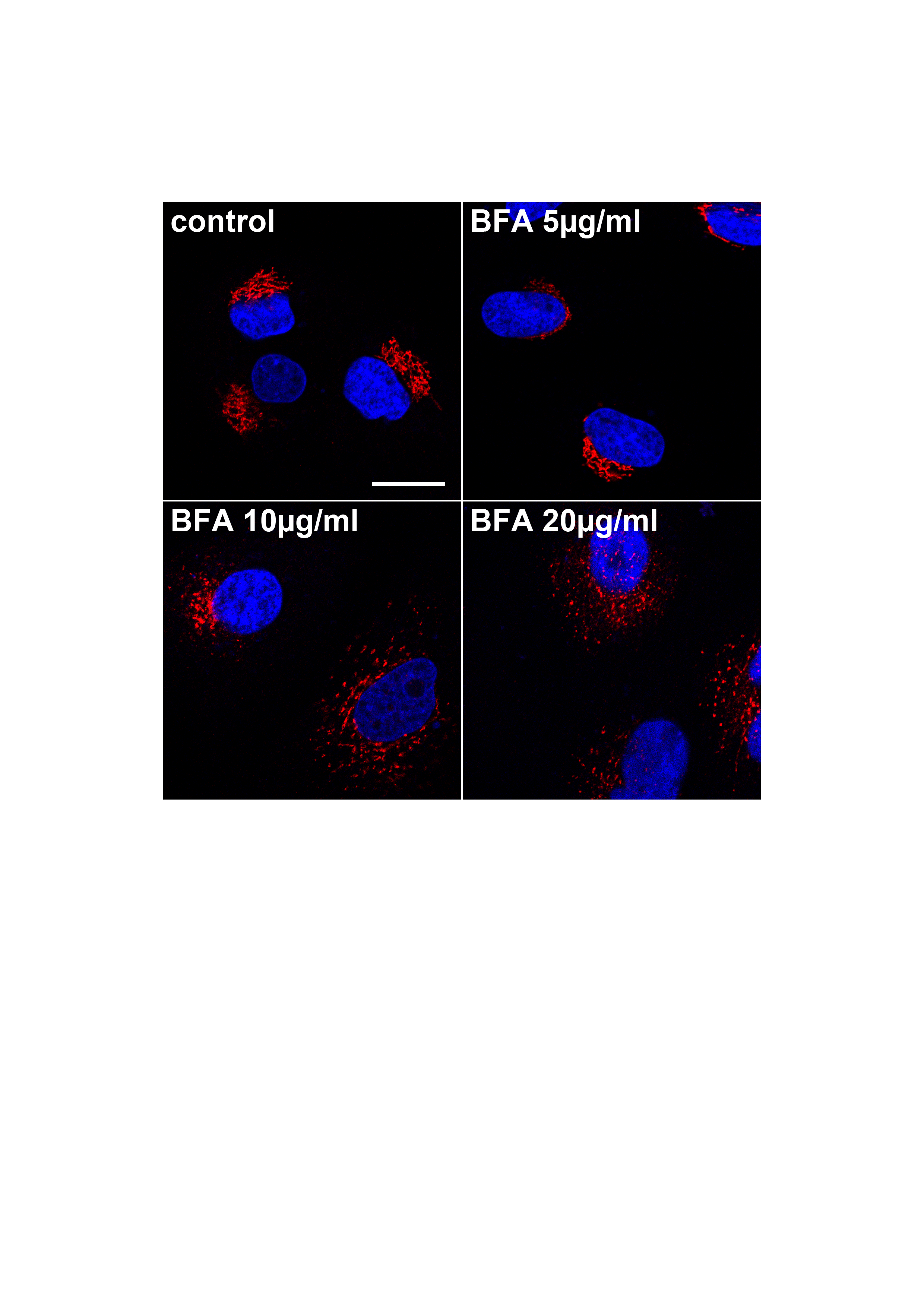

Supplement: Figure S2 — Impact of BFA on the cis-Golgi morphology of MDCK cells. MDCK cells were treated for 30 minutes with the indicated concentrations of BFA, fixed and processed for the immunofluorescent detection of GM130 (shown in red). The nuclei were stained with DAPI (shown in blue). Representative confocal images are presented. Bar, 20 µm. (TIF) [file pone.0074491.s002.tif]
