# Supplementary material for: Hepatitis C Virus Replication and Golgi Function in Brefeldin A-Resistant Hepatoma-Derived Cells
Source: PLoS One. 2013 Sep 18;8(9):e74491. doi: 10.1371/journal.pone.0074491 (PMC3776844; doi:10.1371/journal.pone.0074491)
Supplement: Table S1 — Primers used for GBF1 sequencing. (DOCX) [file pone.0074491.s003.docx]

Table S1. Primers used for GBF1 sequencing

| Primer | Sequence (5’-3’) |
| --- | --- |
| RT1 | GTGGCAGCAACAGCAGCTCT |
| RT2 | GAGAGCAGCACCTGAGCACT |
| RT3 | TGGACAGCAGCTTTGTGAGT |
|  |  |
| 1 FOR | TGCCACCTATGACTCTGCCC |
| 2 FOR | CATGCACGCAGGCTCCAGCG |
| 3 FOR | CATACGGTGTCTTTACAGGT |
| 4 FOR | AGGTCTACACTGACCATGGC |
| 5 FOR | TTTCTGCCTAGAGATGCTGC |
| 6 FOR | AGCAGTGAGTCTATTGAGAA |
| 7 FOR | ATGCCTGCTTTTCCCTGGCC |
| 8 FOR | AGAACATGGGAAATCAGGAT |
| 9 FOR | CGAGAGCACCTCAAGTTCCA |
| 10 FOR | CAGAGCCTGGAAGCAGTGAG |
| 11 FOR | TGAATCTGTGTGTGAGATTA |
| 12 FOR | AGGCCCCGCCATTTTGGATC |
|  |  |
| 1 REV | AGTGGGGTCAAGGAAAGCCT |
| 2 REV | CGTAGGTGAGGGAGAAAACA |
| 3 REV | CAGTAAAGGGCACCAGCAGT |
| 4 REV | CCAGTCCCACTGTTAGGCTG |
| 5 REV | CTCCGGAGAAGCCGAATGGC |
| 6 REV | AGCCTTGGGCAGTAGTTGGG |
| 7 REV | TGATGGCATGGTACATGTCC |
| 8 REV | TTCTGATTGAACTGCTCTGT |
| 9 REV | GTAGTCACAATCATAGTTGA |
| 10 REV | GATTGACGTAATCCATGTCA |
| 11 REV | TTCTTCATGTTGGTCCCCAC |
| 12 REV | TAGATGACCGAAACTATGCA |
